# When communicating health-related knowledge, beware of the black holes of the knowledge landscapes geography

**DOI:** 10.3325/cmj.2016.57.504

**Published:** 2016-10

**Authors:** Srećko Gajović, Anna Lydia Svalastog

**Affiliations:** 1 University of Zagreb School of Medicine, Croatian Institute for Brain Research, Zagreb, Croatia *srecko.gajovic@hiim.hr*; 2 Østfold University College, Department of Health and Social Studies, Fredrikstad, Norway

## Communicating health in the knowledge landscapes

Health-related knowledge is a complicated mixture of levels and dimensions including molecular research, clinical research, well established practices, new technologies and treatments, specialized and individualized life-style recommendations, and quality of life factors. As it concerns everything from microbiology to social well-being, this complex landscape of knowledge is difficult to grasp even by medical professionals. Despite its complexity, the relevance of this knowledge is obvious for the individuals, their families, their professional environment, and the society as a whole. Understanding health-related knowledge is a prerequisite for patient-centered medicine, and being acquainted with the newest developments can imply better health, higher quality of life, and better medical treatment (1,2).

Making healthy decisions is quite a challenge, due to sophisticated and constantly changing status of medical knowledge. For example, regarding dietary requirements, foods that are considered healthy today might be defined as not so healthy tomorrow (as with the Acrylamide debate a few years back), and the prevention activity supported now, could not be recommended tomorrow (as with running being good or hazardous to back bone problems) (3,4). Sometimes it borders on the trivial, eg, whether you should choose an electric tooth brush or not, whether to spend more money on the organic grown food or not (5). But the decisions about health are not trivial; they can substantially influence our lives, and in some cases even they can make a difference between life and death. Therefore, the public interest in health-related knowledge is high and growing, and the question of how to find relevant health-related information gets increasingly important, not only to the acutely or terminally ill persons.

In order to achieve efficient knowledge communication between different stakeholders, we have recently suggested knowledge landscapes as a concept that embraces the present society as a digital and global society, and we have suggested navigation through the landscapes as a concept that embraces the individuals in search of knowledge (6,7). It conceptualizes both successful communication of relevant health advice, as well as impasses and misunderstandings leading to inappropriate health decisions. Instead of using a one-way model to describe communication from experts toward users, and instead of a dialogue model between these two sides, we have suggested a multilateral communication model of different stakeholders. Due to new software (interactive/Web 2.0 programs) and personalized services (algorithms), the formerly separated knowledge traditions meet in the digitalized internet-based frame of the present society.

Digital society drags us all into a constant negotiating of meaning, not the least regarding questions like “What is health?”, “What does a particular diagnosis mean?”, or “What is the best treatment?”. From the perspective of communicating health and medicine in the digital environment, there is a renewed need to clarify the subjects of interest from different angles and perspectives. These angles and perspectives concern both the knowledge and an additional important parameter – the context (values, culture, etc). So does knowledge communication, which needs the context to reveal the meaning. As such, knowledge communication and understanding is context dependent, and we argue that considering the importance of the context is a prerequisite for successful navigation through knowledge landscapes. In this paper we wanted to extend our views of health-related knowledge landscapes by presenting their geography and discussing their dynamics.

## The geography of knowledge landscapes in the digital realm

Multilateral communication that includes multiple participants can occur in both off-line and online settings. Multilateral online interactions are increasingly important as a location where one can get access to information, knowledge, and social surroundings, making the digital realm a major contemporary tool for knowledge dissemination.

The technological advances in the digital realm have provided the necessary platforms that allow knowledge landscapes to form. Those technological advances excel particularly in multilateral communication, and they include social networks (eg, Facebook), blogs and miniblogs (eg, Twitter), forums, virtual networks, and video sharing platforms (eg, YouTube). The internet also provides the tools for classic one-way communication, where contents are collected and systematized in the form of web pages, repositories, and archives, or for a dialogue, enabled through e-mails, instant messaging, or chat services (6).

Specifically in the medical field, the majority of books, scientific journals, and teaching materials have their digital versions. The Open Access approach for scientific information allows every user regardless of education level or affiliation to access original research publications (8). Together with the repositories of books and online courses, large quantities of relevant knowledge, including medical knowledge, can be accessed in the digital world. But the mere presence of this content does not mean that it will be accessed, and if accessed, that it will be understood. Moreover, how the accessed knowledge affects the user depends highly on the context of the knowledge source, as well on the context of the user. Subsequently, the digital knowledge landscapes geography is defined not only as a series of internet locations, but as a complex combination of information, its presentations, user-technology interactions, and the surrounding context.

Academic and research based knowledge is scattered in the digital realm within a variety of virtual domains and frequently hidden. To find it, the users rely on search engines and help each other to find what they judge useful. Unless one searches directly in academic databases (like PubMed, pointing to the research articles, which are in many instances “pay per view”), the results from general online searches using Google or Bing will combine quite different sources, which do not need to be directly related to relevant or evidence-based knowledge. The search results also differ between two users searching exactly the same, because they are formed by the algorithms of a particular search engine that organize and present the information, and by the individual search history as a preference (9).

## The flexibility of knowledge landscapes

The geography of knowledge landscapes is neither flat nor stable. Knowledge is not an absolute, but a tentative entity, therefore its relevance is constantly changing and it is constantly re-evaluated. The temporal dynamic and context dependency is an inherent feature of all traditions claiming its content to be knowledge. The tentativeness of knowledge could be elaborated as its timeliness, dependence on language and culture, or as a carrier of politics (10-12). Both new points of view and innovations are needed to facilitate re-evaluation and developments that anew can make knowledge relevant for particular and tentative contexts. Thus to make knowledge absorbed by society, we need it to be communicated to the users in order to be effectuated, discussed, and publicly inquired.

The flexibility of the knowledge landscapes to incorporate an incoming new knowledge and to adapt to the changes is an asset, and it is enabled by its digital nature. This provides potentially important benefits for the users, which expect to find on the internet the latest version of medical knowledge, or hope to be the first to catch the game changing information of their interest. It is also important for the innovators and the professionals to have a forum to spread and exchange their ideas and to facilitate implementation, eg, of new approaches for the better health care. This dynamic also works the other way around and represents a significant risk of knowledge landscapes distortion by undermining reflexive processes that ensure context sensibility, and by avoiding the use of precautionary principles and implementation of ethics (13).

## Up and down the knowledge landscapes

Digital technology allows for time-dependent changes, by constantly adding new and modifying old contents. But as the landscape constantly changes, it makes navigation through the knowledge landscapes more complicated. The landscape contains layers of old and new, valid and obsolete, just discovered or forgotten. The journey through knowledge landscapes is therefore also a journey in time, and not only an exercise of multilateral communication.

Together with time, as a fourth dimension, the knowledge landscapes’ three-dimensional geometry implies that the view is frequently obstructed, eg, by the mountains and slopes of the digital landscapes. If the landscape was a two-dimensional flat surface, the visibility of the landscape elements would be theoretically without limits and it would be easy to point out directions so that the users could reach what they searched for. But finding online knowledge, understanding it, and using or refusing it is a context-dependent process. Contexts of relevance here are those contexts that work as a carrier of meaning and are inseparable from the users' understanding of a search result. Examples might be a text (eg, a health recommendation already know to the user), a phenomenon (as a disease, an epidemic in past or present time), or a health strategy (for example regarding what is understood as proper hygiene). Thus all contexts are not important in all situations, and to understand how online search results are interpreted, one needs to figure out which contexts are relevant for concrete problems and communicative situations.

What we are saying is that context is fundamental to the interpretation of the communicative situation and it needs to be approached as a key element in the communication process. The myriad of formative contexts includes economy, geography, language, gender, class, sub-cultural belonging, faith and values, as well as political events, experiences from the past, actual recourses at hand, social organization as well as organizational structure and infrastructure, local knowledge traditions (like traditional medicine and healers), media coverage as well as rumors and propaganda. In addition, technology, in particular digital technology, needs to be included as a formative context for communication in a digitalized society. Regarding communication of health and medicine a key challenge is to identify which context is important for dissemination and communication to create transparency and avoid misunderstandings and misconduct.

The context is an integrated part of health-related decision-making, moreover it is also very personal due to the intimate nature of health and disease. For example, the overwhelming majority of people would agree that smoking causes lung cancer, and sex without protection might end in an unwanted pregnancy. Still, the decision whether to smoke or not, whether to use a condom or not, is not only evidence-based, but based on a variety of factors influencing the individual behavior. The results of any health intervention (eg, anti-smoking or contraceptive campaign, preventing infections, or vaccinating children) do not depend only on the evidence-based data, but on understanding and adapting to the particular contexts (14). Present digitalized society is just as context dependent as the society was in earlier times. The new aspect of the global range of digital communication is its immediacy/high speed, as it was recently shown during the latest Ebola outbreak (15).

## The shaping forces of knowledge landscapes geography

The knowledge landscapes geography primarily integrates these two key elements: knowledge and context. The ups and downs in the landscapes (z-dimension in the coordinate system) are shaped by contexts integrated into the digital realm: a) technology, the device and software in use, b) the user and the user’s context, c) the frames and results that are produced through the interaction between technology and user (Table 1). The gravity forces of contexts are thus 3-fold: technological, social-cultural, and the result of the interaction between the first two. The contexts influence the position of the knowledge in the landscapes, and appear as a gravity force shaping the landscape geography. Thus, the knowledge could be hidden, eg, behind a mountain reef representing a language barrier, or due to an algorithm that constantly gives priority to certain results, or due to the way the algorithm reflects past history of the users' search pattern. In consequence, the users (eg, crawling through the narrow canyon of the knowledge landscape) might lose the possibility to see the bigger picture and its framing structure. This visual interpretation of knowledge landscape geographies aims to help us understand the user’s behavior. Moreover, it opens up possibilities for us to get a vocabulary so that we can in a new way discuss how to position the knowledge to be approachable by the users.

**Table 1:** Elements of the knowledge landscapes geography

Knowledge landscapes coordinates	As the knowledge landscapes depict a 3-dimensional space, with time as the 4th dimension, the x-, y-, and z-coordinates apply. Determining what influences the coordinate values is still open for discussion, but we suggest that up and down (z-value) is context dependent, providing a visualization of the knowledge-context interactions. Contexts of relevance are: a) the technology, b) the social, cultural, economic, political, etc, context that contributes meaning to the user’s interpretation of search results, c) the constant production of results from the interaction between technology and user.
Gravity forces	The context is a gravity force that shapes the landscapes, which again host the knowledge (by assigning the z-value to the 3D-space, the context creates mountains, valleys, and other possible geographies).
Centers of gravity	These are coherent and concentrated contexts supported by matching knowledge. They dominate by their gravitational forces in the landscape. They could represent, eg, a university creating a geographical basin around rivers of knowledge, or a conspiracy theory creating a black hole.
Isolated landscapes	They are geographies distorting the knowledge. Isolation results in knowledge being determined and self-confirmatory, lacking tentativeness and re-evaluation.
Black hole	The extreme form of landscape’s geography, which self-perpetuates the distortions and engulfs the surroundings by the force of the gravity center. Represents a social disease.

The Open Access notion, which makes all knowledge digital, available, and free is important as it provides the content to be present on the internet. When we combine knowledge and context, as in the knowledge landscapes, providing content on the internet is just a first step toward sharing knowledge and making it useful. The output of the knowledge communication is context dependent, and the context dimension of the knowledge landscapes will influence what is disseminated and how it is interpreted. Therefore, our understanding of the knowledge landscapes is a tool that helps us understand the complexity of contexts in digital society, as well as helps us to shape the digital technology and assist the users in their navigation toward knowledge and the solutions they search for.

## Black holes as a specific geography of knowledge distortion

Besides being the places of the efficient presentation of academic and research based knowledge, the knowledge landscapes are also the places where knowledge is distorted and where incorrect and misleading information is distributed. The freedom to post diverse contents on the internet, and the egalitarian nature of the internet, where experts are in the same positions as all other content providers, does not allow users to discriminate not relevant from relevant and unreliable from reliable information. Moreover, contents with commercial motivation are frequently scattered among other contents, without clear borders. Therefore, navigation in the digital realm is filled with challenges. There is no objectivity – in the sense of a common organization of the subjects available online. When accessing the internet, the search engines provide us with a personalized adjusted approach, trying to guess what we are searching for (16). Being surrounded by our personal contexts, and guided (or misguided) through internet by search algorithms can bring us to different knowledge landscapes as well as to different positions in relation to knowledge. To facilitate healthy decisions, the ideal landscape’s geography is characterized by openness and flexibility, and allows confronting contents. This enables the user to create her/his own standpoints (both open and flexible) and is a prerequisite for the person-centered medicine.

Knowledge distortions can occur when contrary to open and flexible landscapes, users are isolated in the digital environment (eg, being in a valley surrounded by mountains). The isolation might happen just by accident, reflecting personal search history, interests, and knowledge. It can also be a choice reflecting a conviction or social identity that represents a particular and outspoken stance regarding eg, vaccination, blood transfer, or dietary patterns.

What might at first be an obstacle, or a steep downhill in the knowledge landscapes, can, if repeatedly being visited by the user, work as an undermining and altering force. The location’s relation to other parts of the web, and the algorithms' way of using a preferred site, can affect future search results. In the vocabulary of Michel Foucault, one might say that search choices are transformed by algorithm preferences to search results that create a particular “webs of meanings” (17). The interaction between users and software will transport the users into already established discourses, and it will also create new online “webs of meaning,” which are adjusted to the individual users’ preferences, webs that are wrapping itself around the users and shaping their life and identity. As they may represent counter academic and counter science information, they attain power and potency that can harm the user. The user’s search for knowledge becomes distorted, and what is being presented as search results can be described as a counter discourse. Although Michel Foucault’s vocabulary is powerful (and seductive) in describing the dialectical discourse-counter-discourse movement, this only explains some aspects of how communication is conducted and meaning is constructed online (18). Distorted evidence mostly work as a force on its own terms, ie, not as a dialectical process, and through the repetitive selection of the one-sided contents they act as a gravity force distorting the digital landscape.

Though misconception and distortion might start out as a counter discourse, they may end up as a self-confirmatory process that create a self-perpetuating context, which serves as a new center of gravity, distorting the knowledge landscapes. The information, which confirms the claims of the gravity center appears as stronger than that coming from the outside. Subsequently, the segregation of the content sources occurs and the self-confirmatory content gets assigned a higher value of reliability and trust, which creates the isolated form of knowledge landscapes (Table 1). For example, people in fear of well-established vaccination for children’s diseases like measles are located in a particular isolated landscape, belonging to a group that shares and discusses various internet contents, which are then discussed among them. The gravitational forces shape in a self-confirmatory way the isolated landscape as a valley with the slippery slopes where individual could slip toward twisted understandings and harmful decisions. Still the isolation per se does not determine what is wrong or right, and the isolated groups bound together by gravity forces of the isolated knowledge landscapes could be completely right. Therefore, the topics (ie, centers of gravity) that lead to isolated landscapes are controversial and difficult to handle. It is important to notice that regardless of whether it is wrong or right, the knowledge in the isolated landscape is indeed distorted, as it loses its tentative attribute, to be challenged and re-confirmed. If the communication routes out of the isolated knowledge landscapes remain open, then these could be used to break the isolation and to regain the necessary multilateralism of the knowledge communication.

If the isolation deepens, and the gravity center relying on the isolation gets stronger, the extreme form of knowledge landscapes is forming, which we refer to as a black hole. The gravity forces of the contexts, strengthened by the technology-user interaction, segregate the information sources completely and only those contexts shared within the black hole would be considered relevant. The self-confirmatory and repetitive nature of the information flow isolates the group of individuals in the black hole, within their own dynamic, which is not any more influenced by the outer world. The outside information is per se considered unreliable and even malicious, and attacks and attempts to pull and incorporate additional landscapes into the black hole can be expected. Examples of black holes in knowledge landscapes are regrettably numerous, for example in the medical field it could be avoiding child disease vaccination due to fear of autism, or avoiding chemotherapy due to conspiracy theories. Their characteristics are not too different from other conspiracy theories on the internet, eg, on chemtrails or on water fluoridation. Black holes of the digital realm represent a new form of a social disease, analogous to medical diseases, and it is important to understand them and to find ways to counter them in the future.

Presenting the black holes as a product of an extreme form of isolated knowledge landscapes could hopefully improve the approaches on how to deal with the corresponding topics and social groups. As knowledge landscapes uplift the relation between knowledge and context, they provide geographies that potentially might tackle these social challenges and also strategically approach the isolated landscapes. The openness and flexibility of the digital realm facilitates individual decision and patient autonomy, hence if we could discuss the dynamics behind, processes involved, and bring the contents distributed in isolated landscapes and the black holes to the open, this could profoundly change key premises of the situation.

Isolated landscapes are the products of a process where technological, sociological, and cultural factors merge. Visualizing them creates awareness on how processes, imbedded in digitalized society, are formative for individual health decision makers, affecting the health communicators’ ability to establish communication with users/target groups. This is an urgent matter in a situation where digitalization of health information and communication have become key strategies for governing bodies as well as health institutions and health related non-governmental organizations. In this vein, we indicate that the knowledge landscape concept supports the patient autonomy and person-centered approach in medicine. The importance of context is an argument for the fundamental importance of an interdisciplinary approach in health interventions that aim toward better health.
